# Defective efferocytosis by aged macrophages promotes STING signaling mediated inflammatory liver injury

**DOI:** 10.1038/s41420-023-01497-9

**Published:** 2023-07-08

**Authors:** Haoran Hu, Xuyu Cheng, Fei Li, Zhu Guan, Jian Xu, Dongming Wu, Yiyun Gao, Xinyu Zhan, Ping Wang, Haoming Zhou, Zhuqing Rao, Feng Cheng

**Affiliations:** 1 Hepatobiliary Center, The First Affiliated Hospital of Nanjing Medical University; Key Laboratory of Liver Transplantation, Research Unit of Liver Transplantation and Transplant Immunology, Chinese Academy of Medical Sciences; NHC Key Laboratory of Living Donor Liver Transplantation (Nanjing Medical University), 210029 Nanjing, Jiangsu Province China; 2 Department of Breast Surgery, The First Affiliated Hospital of Nanjing Medical University, 210029 Nanjing, Jiangsu Province China; 3 Department of Anesthesiology, The First Affiliated Hospital of Nanjing Medical University, 210029 Nanjing, Jiangsu Province China

**Keywords:** Mechanisms of disease, Cell death and immune response

## Abstract

Aged livers have shown aggravated liver ischemia and reperfusion (IR) injury. Timely efferocytosis of apoptotic cells is a key mechanism for avoiding excessive inflammation and tissue injury. Here, we investigated the alteration of efferocytosis by aged macrophages and its role in regulating macrophage STING (stimulator of interferon genes) signaling and liver IR injury. Aged and young mice were subjected to liver partial IR model. Liver injury and inflammation were measured. Efferocytosis by aged macrophages and the underlying regulatory mechanism were analyzed as well. Aged macrophages exhibited impaired efferocytosis with decreased MerTK (c-mer proto-oncogene tyrosine kinase) activation, which was reversed by treatment of the MerTK CRISPR activation plasmid. Increased MerTK cleavage by ADAM17 (a disintegrin and metalloproteinase 17) due to enhanced ROS (reactive oxygen species) levels contributed to defective efferocytosis by aged macrophages. MerTK activation by suppressing ADAM17 or ROS improved aged macrophage efferocytosis, leading to reduced inflammatory liver injury. Moreover, increased apoptotic hepatocytes, DNA accumulation, and macrophage STING activation were observed in aged ischemic livers. Improvement in efferocytosis by aged macrophages via MerTK activation suppressed STING activation and inflammatory liver injury. Our study demonstrates that aging suppresses MerTK- mediated macrophage efferocytosis to promote macrophage STING activation and inflammatory liver IR injury, suggesting a new mechanism and potential therapy to promote inflammation resolution and efferocytosis in aged livers.

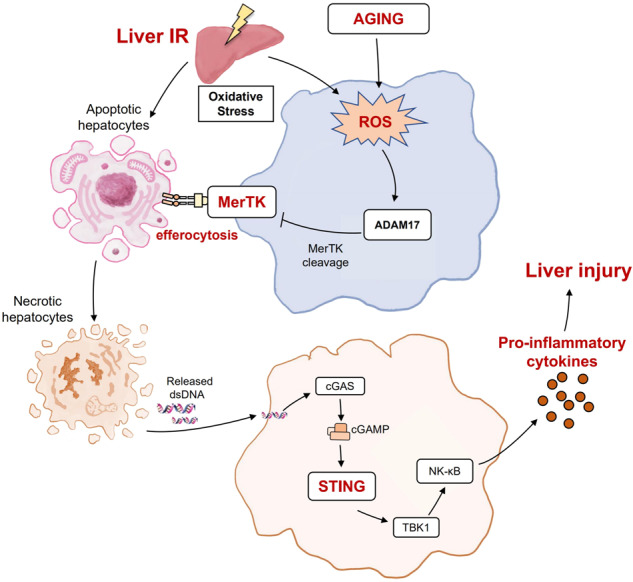

## Introduction

The timely removal of apoptotic cells plays an important role in nearly all tissues during development, homeostasis, and disease. Efferocytosis is the process of engulfing and clearing dead and dying cells by professional and nonprofessional phagocytes, such as macrophages, dendritic cells, and epithelial cells [1]. While efficient efferocytosis promotes inflammation resolution, defective efferocytosis leads to the accumulation of apoptotic cells, secondary post-apoptotic necrosis, and ultimately, increased inflammation [2]. Modulation of efferocytosis is expected to be an effective treatment for various diseases [3].

Liver ischemia and reperfusion (IR) injury is a clinically significant process that occurs during liver trauma, partial hepatectomy, and transplantation, and impairs liver function and patient recovery. The interplay between liver parenchymal cells and non-parenchymal cells, such as macrophages, plays a critical role in regulating liver IR injury [4, 5]. Damage-associated molecular patterns (DAMPs) in stressed hepatocytes activate macrophages via pattern recognition receptors (PRRs), which in turn aggravate inflammatory injury in hepatocytes [6]. Aged livers are more susceptible to IR injury due to dysregulated inflammatory responses, energy metabolism, and autophagy [7]. Aged macrophages exhibit decreased phagocytosis and increased production of inflammatory cytokines [8].

The cGAS(Cyclic GMP-AMP Synthase)-STING signaling pathway has emerged as a key mediator of inflammation during infection, tumors, inflammation, and autoimmune diseases [9]. In addition to microbial DNA, endogenous DNA from nuclear chromatin and mitochondria can also be detected by cGAS. DNA damage and inflammation are the hallmarks of age-related diseases [10]. Previously, we found that aging aggravated liver IR injury by enhancing the STING-NLRP3(NOD-like receptor thermal protein domain associated protein 3)-mediated pro-inflammatory response of macrophages [11, 12]. The TAM family of receptor tyrosine kinases (Tyro3, Axl, and MerTK) plays an essential role in the regulation of efferocytosis [13]. Interestingly, a recent study demonstrated that blocking MerTK suppressed the clearance of apoptotic tumor cells and promoted macrophage STING activation [14]. Protective effects of efferocytosis by macrophages have been reported to promote inflammation resolution by timely clearing of apoptotic cells after IR insult [15–17]. However, the alteration of efferocytosis by aged macrophages and its role and underlying mechanism in regulating macrophage STING activation during liver IR injury remain unclear.

Here, we investigated the role of efferocytosis in regulating the accumulation of apoptotic hepatocytes and subsequent extracellular released DNA in activating macrophage STING signaling. Decreased MerTK activation was observed in aged macrophages, leading to defective clearance of apoptotic hepatocytes by macrophage efferocytosis. Enhanced production of ROS suppresses MerTK activity by promoting MerTK cleavage via ADAM17. Our findings suggest an interplay between efferocytosis and STING activation in macrophages, providing a potential target for the intervention of liver IR injury.

## Results

### Aging suppressed efferocytosis of apoptotic hepatocytes by macrophages

**Fig. 1:**
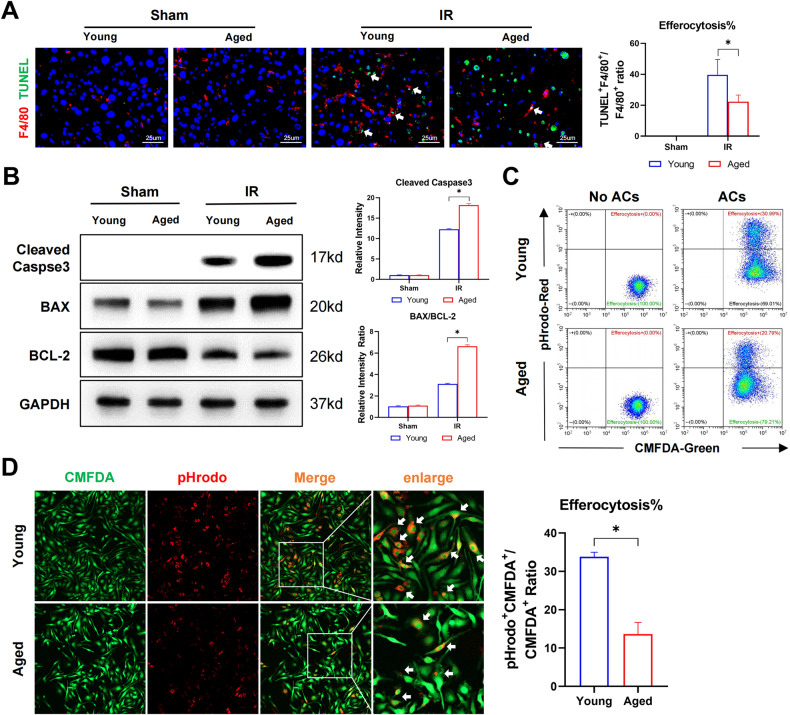
Aging suppressed efferocytosis of apoptotic hepatocytes by macrophages. **A**, **B** Young and Aged C57BL/6 mice were subjected to liver IRI as the methods described. (*n* = 6–8). **A** Representative F4/80 (Cy3) and TUNEL staining of liver section as described in situ efferocytosis in methods. Typical efferocytosis were marked with white arrows. **B** Young and Aged mice liver Cleaved-caspase 3, BCL-2, BAX, and GAPDH were detected by western blot. Data are presented as the Mean ± SEM. **C**, **D** Young and aged BMDMs labeled with CMFDA-green were incubated with apoptotic cells labeded with pHrodo-red, in vitro efferocytosis were detected by flow cytometry (**C**) as well as immunofluorescence photography, typical efferocytosis were marked with white arrows (**D**). All these experiments have been repeated for three times. *P*-value < 0.05 was considered significant.

We first compared efferocytosis of apoptotic cells between young and aged macrophages. In vivo analysis of macrophage efferocytosis was accomplished by quantifying the fluorescence colocalization of TUNEL-positive apoptotic cells and F4/80 positive macrophages in the liver post-IR. Figure 1A shows that IR triggered intrahepatic apoptosis of hepatocytes and infiltration of macrophages, as determined by increased TUNEL+ and F4/80+ positive staining of cells in the liver post-IR. Moreover, fewer TUNEL and F4/80 double-positive staining cells, but many more single TUNEL-positive cells were detected in aged livers after IR. Western blotting also showed increased protein levels of cleaved caspase3 and BAX/BCL-2 ratio in aged livers (Fig. 1B). Macrophage efferocytosis was assessed in vitro by incubating BMDMs (bone marrow-derived macrophage) with apoptotic Jurkat cells. Phagocytosis of apoptotic Jurkat cells by BMDMs was observed at 45 min post co-culture, as shown by red fluorescent apoptotic Jurkat cells (ACs) in green-labeled macrophages detected by FCM (Fig. 1C) and IF (Fig. 1D). Aged macrophages significantly suppressed the phagocytosis of ACs (Fig. 1C, D). These results indicate that aging impaired efferocytosis of apoptotic cells by macrophages, leading to increased accumulation of apoptotic hepatocytes in the liver post-IR.

### Aging impaired macrophage efferocytosis by inhibiting MerTK activation

**Fig. 2:**
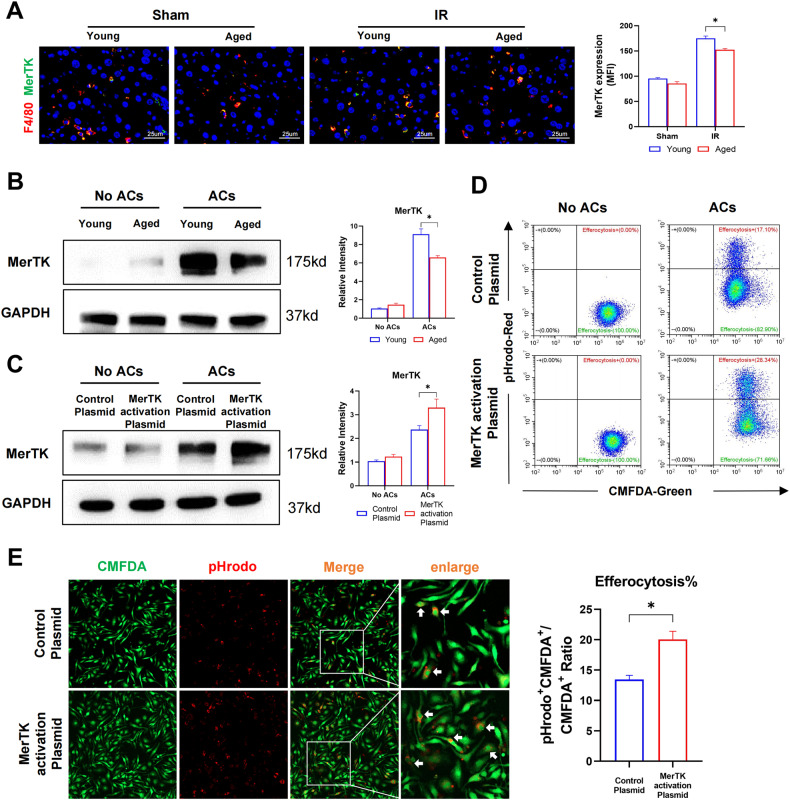
Aging impaired macrophage efferocytosis by inhibiting MerTK activation. **A** Young and Aged C57BL/6 mice were subjected to liver IRI as the methods described. (*n* = 6-8). Representative F4/80 (Cy3) and MerTK (Alexa Flour 488) staining of liver section. **B** Young and Aged BMDMs were incubated with apoptotic cells. MerTK expression in BMDMs were detected by western blot. Data are presented as the Mean ± SEM. **C**–**E** BMDMs were transfected with MerTK CRISPR activation plasmid or control plasmid as methods described. **C** BMDMs were incubated with apoptotic cells. MerTK expression in BMDMs were detected by Western blot. Data are presented as the mean ± SEM. **D**, **E** BMDMs labeled with CMFDA-green were incubated with apoptotic cells labeded with pHrodo-red, in vitro efferocytosis were detected by flow cytometry (**D**) as well as immunofluorescence photography, typical efferocytosis were marked with white arrows (**E**). All these experiments have been repeated for three times. *P*-value < 0.05 was considered significant.

MerTK, a family member of TAM receptors, regulates macrophage efferocytosis [18]. To examine the potential role of MerTK signaling in modulating macrophage efferocytosis, MerTK expression in macrophages was compared between young and aged mice after IR. Indeed, IR triggered MerTK activation of macrophages in the liver post-IR, which was decreased in aged mice (Fig. 2A). Aged BMDMs stimulated with ACs also showed decreased MerTK activation in vitro (Fig. 2B).

Next, we determined the functional significance of suppressed MerTK expression in the regulation of efferocytosis by aged macrophages. Aged BMDMs were transfected with the MerTK CRISPR activation plasmid, and in vitro efferocytosis was evaluated. MerTK activation was confirmed by western blot (Fig. 2C). Restoration of MerTK activation significantly increased the phagocytosis of ACs by aged macrophages, as shown by FCM (Fig. 2D) and immunostaining analysis (Fig. 2E). Therefore, suppression of MerTK activation is responsible for defective efferocytosis by aged macrophages.

### Enhanced cleavage of MerTK by ADAM17 contributed to defective efferocytosis by aged macrophages

**Fig. 3:**
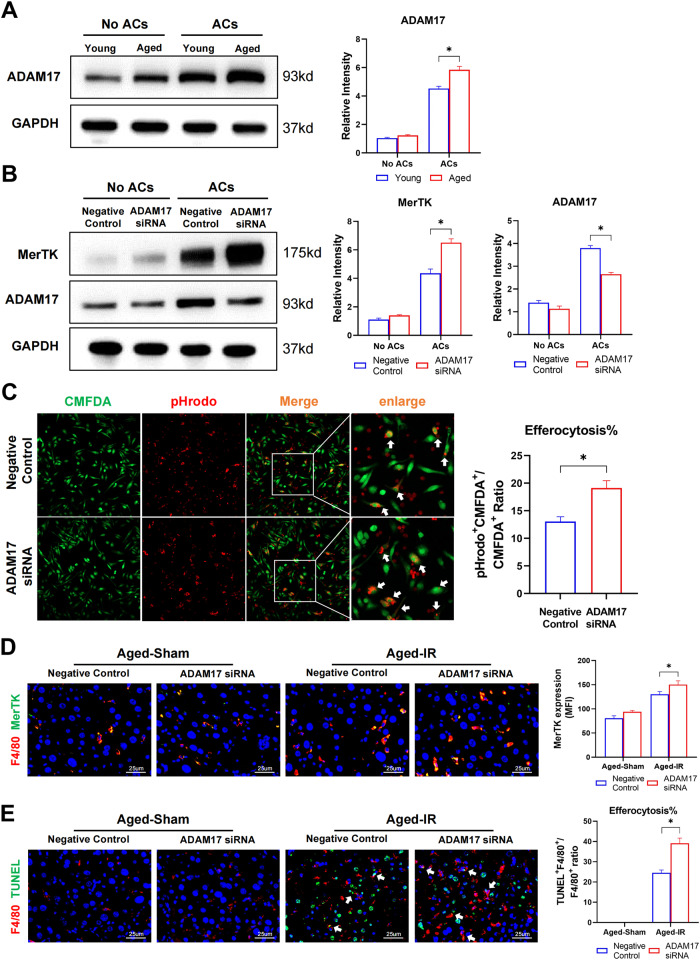
Enhanced cleavage of MerTK by ADAM17 contributed to defective efferocytosis by aged macrophages. **A** Young and Aged BMDMs were incubated with apoptotic cells. ADAM17 expression in BMDMs were detected by Western Blot. Data are presented as the Mean ± SEM. **B**, **C** Aged BMDMs were transfected with ADAM17 siRNA or Negative Control siRNA before efferocytosis. **B** BMDMs were incubated with apoptotic cells. MerTK and ADAM17 expression of BMDMs were detected by Western Blot. Data are presented as the Mean ± SEM. **C** BMDMs labeled with CMFDA-green were incubated with apoptotic cells labeded with pHrodo-red, in vitro efferocytosis were detected by immunofluorescence photography, typical efferocytosis were marked with white arrows. **D**, **E** Aged C57BL/6 mice were subjected to liver IRI after in vivo transfected with ADAM17 siRNA or Negative Control siRNA as the methods described. (*n* = 6–8). **D** Representative F4/80(Cy3) and MerTK (Alexa Flour 488) staining of liver section. **E** Representative F4/80(Cy3) and TUNEL staining of liver section as described in situ efferocytosis in methods. Typical efferocytosis were marked with white arrows. All these experiments have been repeated for three times. *P*-value < 0.05 was considered significant.

ADAM17-mediated proteolytic cleavage is an important mechanism that limits MerTK activity [19]. Next, we examined the expression of ADAM17 in macrophages and found that aged BMDMs showed increased ADAM17 activation after ACs stimulation (Fig. 3A). Furthermore, ADAM17 siRNA was used to suppress the expression of ADAM17 and the proteolytic cleavage of MerTK. The results showed that AMDM17 siRNA restored MerTK activation (Fig. 3B) and promoted phagocytosis of ACs by aged macrophages in vitro (Fig. 3C). In vivo ADAM17 siRNA transfection also enhanced MerTK activation in intrahepatic macrophages in aged mice (Fig. 3D) and promoted efferocytosis of apoptotic cells by aged macrophages after IR (Fig. 3E).

### Aging promoted macrophage ROS production to induce ADAM17-mediated MerTK cleavage and efferocytosis suppression

**Fig. 4:**
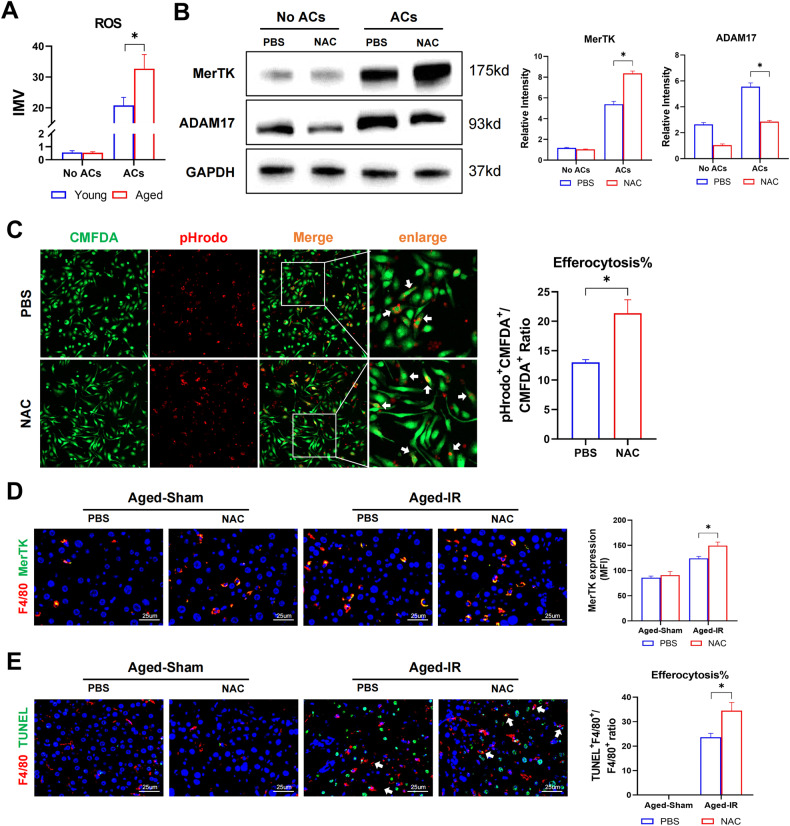
Aging promoted macrophage ROS production to induce ADAM17-mediated MerTK cleavage and efferocytosis suppression. **A** ROS detective of BMDMs before and after efferocytosis. **B**, **C** BMDMs were pretreated with N-Acetylcysteine (NAC), an inhibitor of ROS, or PBS control before efferocytosis. **B** BMDMs were incubated with apoptotic cells. MerTK and ADAM17 expression of BMDMs were detected by western blot. Data are presented as the mean ± SEM. **C** BMDMs labeled with CMFDA-green were incubated with apoptotic cells labeded with PHrodo-red, in vitro efferocytosis were detected by immunofluorescence photography, typical efferocytosis were marked with white arrows. **D**, **E** Aged C57BL/6 mice were subjected to liver IRI after pretreated with NAC or PBS as the methods described. (*n* = 6–8). **D** Representative F4/80(Cy3) and MerTK (Alexa Flour 488) staining of liver section. **E** Representative F4/80(Cy3) and TUNEL staining of liver section as described in situ efferocytosis in methods. Typical efferocytosis were marked with white arrows. All these experiments have been repeated for three times. *P*-value < 0.05 was considered significant.

We previously found that increased oxidative stress in aged macrophages after IR [12] and ROS, which plays a key role of signal transduction [20], have been implicated in modulating ADAM17 activation [21]. Indeed, increased ROS levels were detected in aged macrophages after ACs stimulation (Fig. 4A). ROS scavenging by NAC decreased ADAM17 expression and increased MerTK expression in aged macrophages (Fig. 4B), leading to the enhanced phagocytosis of ACs by aged macrophages in vitro (Fig. 4C).

In vivo, NAC treatment enhanced MerTK activation in macrophages (Fig. 4D) and restored phagocytosis of apoptotic cells by aged macrophages after IR (Fig. 4E).

### Increased apoptotic hepatocytes and DNA accumulation promote macrophage STING activation to induce inflammatory IR injury in aged livers

**Fig. 5:**
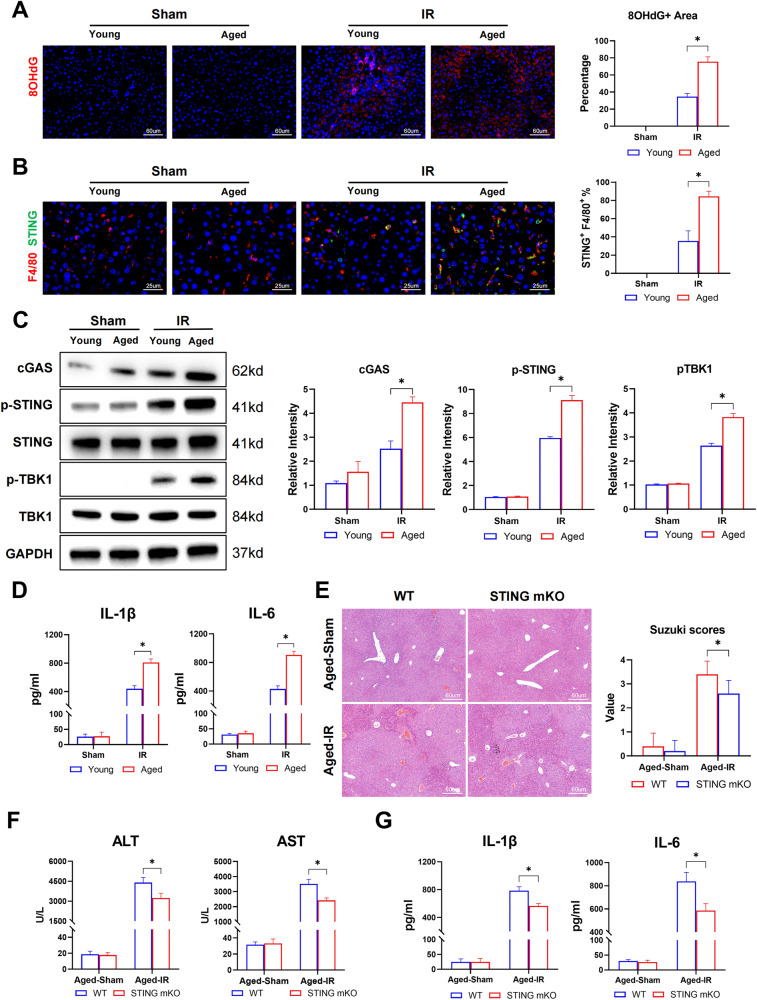
Increased apoptotic hepatocytes and DNA accumulation promoted macrophage STING activation to induce inflammatory IR injury in aged livers. **A**–**D** Young and Aged C57BL/6 mice were subjected to liver IRI as the methods described. (*n* = 6-8). **A** Representative 8-OHdG staining of liver section to show DNA damage. **B** Representative F4/80 (Cy3) and STING (Alexa Flour 488) staining of liver section. **C** Young and Aged mice liver cGAS, p-STING S365, STING, p-TBK1 S172, TBK1, and GAPDH were detected by western blot. Data are presented as the mean ± SEM. **D** ELISA analysis of IL-1β and IL-6 in serum. **E**, **F** Aged WT and STING-mKO mice were subjected to liver IRI as the methods described. (*n* = 6–8). **E** Representative HE staining of liver section. Suzuki Scores were used to assess liver damage. **F** Serum ALT, AST of mice. **G** ELISA analysis of IL-1β and IL-6 in serum. All these experiments have been repeated for three times. *P*-value < 0.05 was considered significant.

The interplay between hepatic parenchymal cell injury/death and macrophage-related inflammation plays an important role in regulating liver IR injury. End-stage apoptotic cells generally represent a necrotic morphotype that induces pro-inflammatory responses [22]. Next, we investigated the consequences of increased accumulation of apoptotic cells caused by defective macrophages in aged livers after IR. Immunogenic DNA released from dying hepatocytes was measured by 8-OHdG staining of the livers post-IR. Significantly increased released DNA was detected in aged livers after IR (Fig. 5A). Consequently, increased activation of STING signaling was observed in liver macrophages, as shown by enhanced STING+ and F4/80+ double staining (Fig. 5B). Aged livers also showed increased protein levels of cGAS, p-STING, and p-TBK1 (Fig. 5C), accompanied by increased serum levels of IL-1b and IL-6 (Fig. 5D).

We previously found that STING inhibition or global STING KO alleviated IR injury in aged liver [11, 12]. To further confirm the cell type-specific role of STING signaling in regulating liver IR injury, myeloid STING KO mice were used, and liver IR injury was analyzed. Indeed, myeloid STING deficiency significantly reduced IR injury (Fig. 5E, F) and serum levels of inflammatory IL-1b and IL-6 (Fig. 5G) in aged mice.

### Restoration of MerTK-mediated efferocytosis suppresses STING activation in aged macrophages and liver inflammation post-IR

**Fig. 6:**
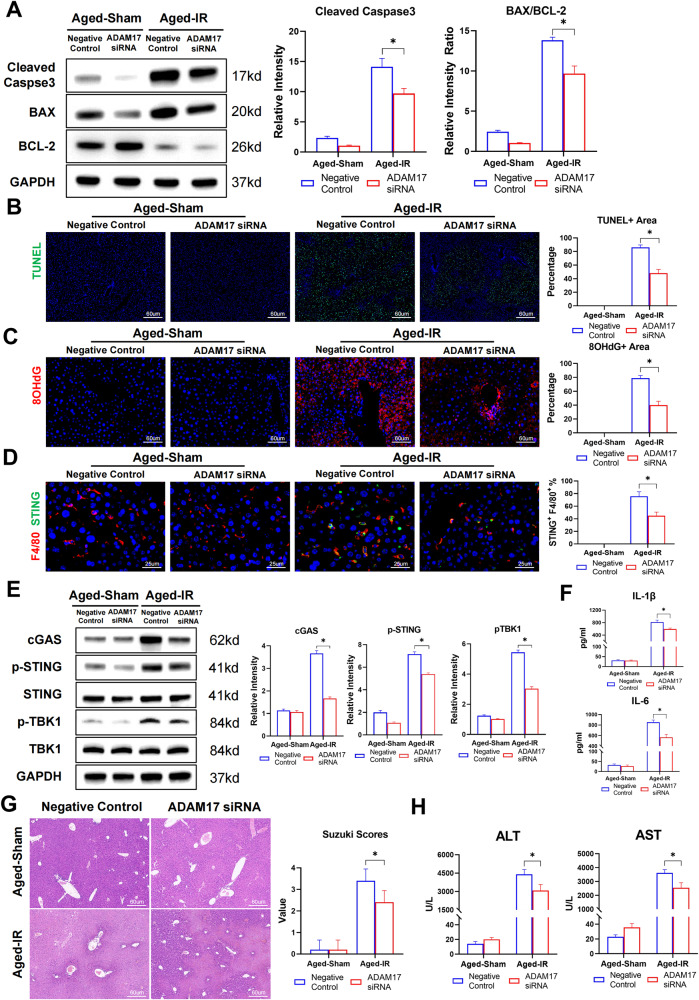
Restoration of MerTK-mediated efferocytosis suppressed STING activation in aged macrophages and liver inflammation post-IR. **A**–**H** Aged C57BL/6 mice were subjected to liver IRI after in vivo transfected with ADAM17 siRNA or Negative Control siRNA as the methods described. (*n* = 6–8). **A** Mice liver Cleaved-caspase 3, BCL-2, BAX, and GAPDH were detected by Western Blot. Data are presented as the Mean ± SEM. **B** Representative TUNEL staining of liver section. **C** Representative 8-OHdG staining of liver section to show DNA damage. **D** Representative F4/80 (Cy3) and STING (Alexa Flour 488) staining of liver section. **E** Mice liver cGAS, p-STING S365, STING, p-TBK1 S172, TBK1, and GAPDH were detected by western blot. Data are presented as the mean ± SEM. **F** ELISA analysis of IL-1β and IL-6 in serum. **G** Representative HE staining of liver section. Suzuki Scores were used to assess liver damage. **H** Serum ALT, AST of mice. All these experiments have been repeated for three times. *P*-value < 0.05 was considered significant.

To determine whether the increased macrophage STING activation was caused by defective efferocytosis of apoptotic cells by aged macrophages after IR, aged mice were transfected with ADAM17 siRNA to restore the MerTK-mediated efferocytosis. The results showed that MerTK activation by ADAM17 siRNA protected aged livers against IR injury, as evidenced by better preserved liver architecture with decreased cleaved caspase3 and BAX/BCL2 ratios (Fig. 6A) and fewer TUNEL-positive areas (Fig. 6B).Moreover, the results showed that ADAM17 siRNA transfection reduced DNA release from dying liver cells (Fig. 6C), leading to decreased activation of cGAS-STING signaling in aged macrophages, as shown by immunostaining and immunoblotting analyses (Fig. 6D, E). Aged mice treated with ADAM17 siRNA also showed decreased levels of serum IL-1b and IL-6 (Fig. 6F).

The results showed that MerTK activation by ADAM17 siRNA protected aged livers against IR injury, as evidenced by better preserved liver architecture with lower Suzuki scores (Fig. 6G) and lower levels of serum ALT and AST (Fig. 6H).

**Fig. 7:**
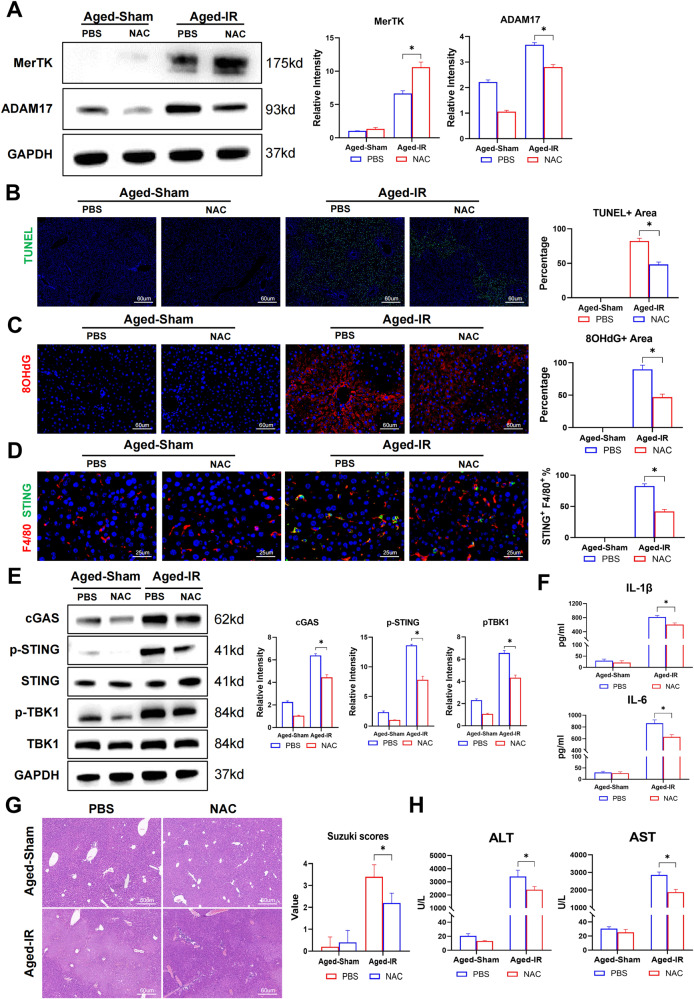
Efferocytosis restoration by NAC suppressed STING activation in aged macrophages and inflammatory liver IR injury. **A**–**H** Aged C57BL/6 mice were subjected to liver IRI after pretreated with NAC or PBS intraperitoneal injection as the methods described. (*n* = 6–8). **A** Mice liver MerTK and ADAM17 were detected by western blot. Data are presented as the mean ± SEM. **B** Representative TUNEL staining of liver section. **C** Representative 8-OHdG staining of liver section to show DNA damage. **D** Representative F4/80 (Cy3) and STING (Alexa Flour 488) staining of liver section. **E** Mice liver cGAS, p-STING S365, STING, p-TBK1 S172, TBK1, and GAPDH were detected by western blot. Data are presented as the mean ± SEM. **F** ELISA analysis of IL-1β and IL-6 in serum. **G** Representative HE staining of liver section. Suzuki Scores were used to assess liver damage. **H** Serum ALT, AST of mice. All these experiments have been repeated for three times. *P*-value < 0.05 was considered significant.

Similarly, in vivo NAC pretreatment also promoted MerTK activation by suppressing ADAM17 activation (Fig. 7A), leading to fewer TUNEL-positive areas (Fig. 7B), suppressed DNA release (Fig. 7C) and subsequent macrophage STING signaling activation (Fig. 7D, E), inflammation (Fig. 7F) and attenuated liver IR injury (Fig. 7G, H) in aged mice after IR. These findings demonstrate that aging suppressed MerTK-mediated macrophage efferocytosis to promote macrophage STING activation and pro-inflammatory responses during liver IR injury.

## Discussion

In the present study, we found that defective macrophage efferocytosis aggravates liver IR injury in aged mice. Suppression of ROS-ADAM17-MerTK signaling-mediated efferocytosis of apoptotic hepatocytes by macrophages caused an increased release of immunogenic DNA from dying hepatocytes, which in turn promoted macrophage STING activation and inflammatory injury in aged livers. Restoration of macrophage efferocytosis attenuates liver IR injury in aged mice.

Excessive or sustained inflammation can induce tissue injury and disease. Various types of cell death, such as apoptosis, necrosis, necroptosis, pyroptosis, and ferroptosis, have been revealed to exhibit distinct proinflammatory properties [23, 24]. Both apoptosis and necrosis were observed in hepatocytes after IR [25]. IR induces periportal expression of MLKL in human liver grafts, and is associated with early allograft dysfunction after transplantation [26]. Ferroptosis is induced by iron overload during liver IR injury [27]. We and others have demonstrated that pyroptosis contributes to the pathogenesis of liver IR injury [28, 29]. However, the precise form of cell death upon liver IR injury remains unclear.

In contrast to necrotic cells, apoptotic cells maintain their plasma membrane integrity with immunologically silent properties. However, end-stage apoptotic cells generally show complete breakdown of the plasma membrane and a necrotic morphotype, which causes pro-inflammatory responses [22]. Therefore, the timely clearance of apoptotic cells is critical for tissue homeostasis. Efferocytosis is predominantly performed by macrophages and, to a lesser extent, by other phagocytes, such as monocytes, dendritic cells, and epithelial cells to clear apoptotic cells, which play essential roles in tissue homeostasis, tissue repair, host defense, and organismal health [1, 2]. Aberrations in efferocytosis due to various causes, including overwhelming clearance machinery, disruptions at different stages of efferocytosis, and dysfunction of phagocytes, are associated with numerous inflammatory pathologies.

Efferocytosis by macrophages to clear apoptotic cells plays a protective role in ischemic injury of various organs. Efferocytosis by cardiac-resident macrophages promotes the clearance and degradation of apoptotic cardiomyocytes after myocardial infarction, which facilitates inflammation resolution [15]. MerTK signaling is responsible for efferocytosis by monocytes/macrophages [30, 31], and MerTK cleavage in macrophages compromises repair after myocardial IR injury [32]. Additionally, efferocytosis by macrophages regulates inflammation and tissue injury in the kidney [16] and brain [17]. The regulation of macrophage activation by targeting various signaling pathways can effectively improve liver injury [33–35]. However, relatively few studies have shown the role of macrophage efferocytosis in liver IR injury. TIM-4 was found to be critical for hepatic macrophages in both the activation and resolution of liver IR injury via efferocytosis [36]. Activation of the TAM receptor promotes efferocytosis of apoptotic cells by macrophages and facilitates macrophage M2 polarization [37, 38]. The enhancement of macrophage efferocytosis by resolvin D1 treatment attenuates liver IR injury [39].

Emerging evidence has shown increased intrahepatic inflammation and tissue injury in aged livers, leading to poor clinical outcomes post-transplantation with aged donor livers [40]. Alterations in the inflammatory response, energy metabolism, and autophagy in aged livers are critically involved in aggravated liver IR injury [7]. We recently reported that aging aggravated liver IR injury by enhancing the STING-NLRP3-mediated proinflammatory response of macrophages [11]. Defective mitophagy in aged macrophages increases mtDNA cytosolic release to promote mtDNA-cGAS-STING activation during sterile liver inflammation, including IR [12, 41]. While these studies mainly analyzed the regulatory mechanism of mtDNA in macrophages on STING activation, the effect of exogenous dsDNA, such as dsDNA or mtDNA released from stressed hepatocytes, on the activation of STING signaling in macrophages is unclear. In the present study, we found that defective efferocytosis by aged macrophages increased the number of apoptotic hepatocytes after IR and subsequent DNA release, which in turn promoted STING activation in aged macrophages.

The TAM receptors Tyro3, Axl, and MerTK are a family of receptor tyrosine kinases with shared ligands Gas6 and protein S, which promote efferocytosis and M2 polarization of macrophages [42]. Emerging evidence suggests that TAM receptor tyrosine kinases are important innate immune checkpoints in cancer therapy and other inflammatory diseases [18]. MerTK is highly expressed in macrophages, and its activation is involved in the internalization of apoptotic cells, suppression of inflammation, and synthesis of inflammatory mediators. Cell surface proteolytic cleavage by ADAM17 protease is an important mechanism that limits MerTK activity [19]. MerTK cleavage results in the production of soluble Mer protein, which prevented Gas6-mediated stimulation of membrane-bound Mer, leading to defective macrophage efferocytosis. MerTK cleavage-resistant (CR) mice showed increased circulating levels of pro-resolving mediators and less lung injury after IR [43]. Blockade of MerTK by an antibody promotes the accumulation of apoptotic cells within tumors to induce macrophage STING activation and anti-tumor immunity [14]. It has been reported that aging impaired peritoneal macrophage phagocytosis of fluorescent particles, while the function of efferocytosis of apoptotic cells by young and aged macrophages were not studied [44]. Here, we studied efferocytosis by aged macrophages both in vivo and in vitro, and found that MerTK signaling was suppressed in aged macrophages, resulting in defective efferocytosis of hepatocytes and aggravated inflammatory liver injury. ROS, a common component of liver IR, promotes the proteolytic cleavage of MerTK [21]. Meanwhile, ROS scavenging by NAC prevents p38 MAPK phosphorylation and ADAM17-mediated MerTK cleavage [45]. Consistently, we found that NAC treatment suppressed ADAM17 activation and MerTK cleavage to promote macrophage efferocytosis. Interestingly, the efferocytosis-independent effects of MerTK have also been reported. Macrophage MerTK activation is enhanced in nonalcoholic steatohepatitis (NASH) due to suppression of its cleavage by ADAM17, which activates hepatic stellate cells (HSC) via the ERK-TGFβ1 pathway to promote liver fibrosis [46].

To the best of our knowledge, this is the first study to report the critical role of efferocytosis in regulating macrophage STING signaling and IR injury in aged livers. We demonstrated that aging promotes MerTK cleavage to suppress macrophage efferocytosis, resulting in the accumulation of apoptotic hepatocytes and subsequent enhanced macrophage STING activation. Our results directly link efferocytosis by macrophages to inflammation resolution and tissue injury, and identify ROS-ADAM17-MerTK signaling as a key mechanism in regulating efferocytosis by aged macrophages.

## Materials and methods

### Animals

Young (8 weeks) and aged (100 weeks) C57BL/6 mice were purchased from the Ziyuan Laboratory Animal Technology Co., Ltd. STING myeloid-specific knockout (STING mKO) mice were created by STING FL/FL mice and myeloid-specific Cre (Lyz2-Cre) mice, generated by Shanghai Model Organisms Center Inc., and the STING FL/FL mice were treated as wild-type control mice (WT). The mice were housed in a standard specific pathogen-free environment under a 12 hours light/dark cycle. All animals received humane care and all animal procedures met the relevant legal and ethical requirements according to the protocols (number NMU08-092) approved by the Institutional Animal Care and Use Committee of Nanjing Medical University.

### Mouse liver IRI model

A mature model of partial hepatic warm IRI has been used [12, 33]. Briefly, after successful anesthesia with isoflurane, heparin was injected into the mice. A midline laparotomy was performed, and an atraumatic clip was used to interrupt the arterial and portal venous blood supply to the cephalic lobes (70%) of the liver. After 90 min of partial hepatic warm ischemia, the clip was removed to initiate the process of hepatic reperfusion. Mice were maintained anesthetized with isoflurane and placed in the environment temperature at 26 °C. The mice were killed after 6 h of reperfusion and the collected samples were harvested for analysis. Sham controls underwent the same procedure but without vascular occlusion.

To study the effects of ROS, mice were pretreated with 300 mg/kg N-acetylcysteine (NAC) (Yeasen Biotechnology, Shanghai, China) or PBS by intraperitoneal injection.

Serum ALT and AST levels were measured using an AU680 clinical chemistry analyzer (Beckman Coulter, Brea, California, USA).

Liver specimens were fixed in 4% paraformaldehyde and embedded in paraffin for hematoxylin and eosin (H&E) and immunofluorescent staining. Some of the specimens were frozen in liquid nitrogen for 8-OHdG staining.

### BMDM cultures

Bone marrow cells were flushed from femurs and tibias. After filtration through a 70-μm strainer, the cells were cultured in Dulbecco’s modified Eagle’s medium (DMEM; Gibco, Franklin, Tennessee, USA) supplemented with 10% FBS(Gibco, Franklin, Tennessee, USA), 1% P/S(Gibco, Franklin, Tennessee, USA), 1% HEPES(Gibco, Franklin, Tennessee, USA), and 20 μg/L M-CSF (macrophage Colony Stimulating Factor; PeproTech, Rocky Hill, New Jersey, USA) for 7 days in a humidified CO_2_ incubator at 37 °C. BMDMs were replated in six-well dishes at a density of 5 × 10^5^ or in confocal dishes at a density of 1 × 10^4^ and cultured overnight for further experiments.

### In vitro MerTK CRISPR activation plasmid transfection

In vitro MerTK CRISPR activation plasmid transfection was performed according to the manufacturer’s protocol. Briefly, for each transfection, 2 µg of MerTK CRISPR Activation Plasmid DNA (Santa Cruz Biotechnology, Dallas, Texas, USA) was diluted into 150 µl plasmid transfection medium (Santa Cruz Biotechnology, Dallas, Texas, USA) and incubated for 5 min at 26 °C. At the same time, 10 µl UltraCruz® Transfection Reagent (Santa Cruz Biotechnology, Dallas, Texas, USA) was diluted with sufficient plasmid transfection medium to bring the final volume to 150 µl and stand for 5 min at 26 °C. Plasmid DNA solution was added directly to the diluted transfection reagent, vortexed immediately, and incubated for 20 min at 26 °C. Before transfection, the BMDMs were replaced with fresh antibiotic-free DMEM. The above-mentioned complex (300 µl) was then added dropwise to each well and incubated for 48 h. Controls were subjected to the same procedures as the control CRISPR/Cas9 plasmid (Santa Cruz Biotechnology, Dallas, Texas, USA). After transfection, the BMDM culture medium was replaced for subsequent experiments.

### In vivo ADAM17 siRNA transfection

According to the manufacturer’s protocol, 1 mg/kg ADAM17 siRNA (Thermo Fisher Scientific, Waltham, Massachusetts, USA) with Invivofectamine 3.0 Reagent (Thermo Fisher Scientific, Waltham, Massachusetts, USA) was used for tail vein injection, while the Negative Control siRNA with vector was used as control.

### In vitro ADAM17 siRNA transfection

ADAM17 siRNA was performed according to the manufacturer’s protocol. Briefly, the BMDMs were replaced with fresh antibiotic-free DMEM for 1 day, followed by cultured with fresh serum-free DMEM medium. For each well, 10 pmol ADAM17 siRNA with 250ul serum-free DMEM medium were mixed softly. Then 5 μl diluted Lipofectamine™ 3000 Reagent (Thermo Fisher Scientific, Waltham, Massachusetts, USA) were added and incubated for 5 min. Finally, the complex was added to cells and incubated for 12 hours. After transfection, the BMDM culture medium was replaced for subsequent experiments.

### Induction of apoptosis and fluorescent labeling of jurkat cells

Jurkat cells were obtained from NCACC and cultured in Roswell Park Memorial Institute 1640 medium (RPMI-1640; Gibco, Franklin, Tennessee, USA) supplemented with 10% FBS, 1% P/S, and 1% HEPES. Cells were cultured in a humidified CO_2_ incubator at 37 °C. Cells were irradiated under a 254 nm UV lamp for 15 min, followed by incubation under normal cell culture conditions for 2 h. Apoptotic cells (ACs) were centrifugally washed twice with RPMI 1640, resuspended at a concentration of 1 × 10^6^ cells/ml in PBS, incubated with 20 ng/ml pHrodo-red (Thermo Fisher Scientific, Waltham, Massachusetts, USA) on a shaker, centrifuged twice, and resuspended in DMEM complete medium for co-culture.

### In situ efferocytosis

For in situ efferocytosis [30], fixed liver specimens were blocked for 60 min, then incubated overnight at 4 °C with the anti-F4/80 (1:200), followed by incubation with Cy3-labeled secondary antibodies; TUNEL staining was performed next, and finally counterstained with DAPI. Efferocytosis was measured by counting the percentage of TUNEL-positive F4/80 positive cells in the individual tissue sections.

### In vitro efferocytosis

For in vitro efferocytosis [47], BMDMs and apoptotic Jurkat cells were prepared as described previously. BMDMs were washed twice and incubated with 1 µM CMFDA-green (Yeason Biotechnology, Shanghai, China) in incubators, washed to remove excess dye, and cultured in complete DMEM for 30 min. PHrodo-labeled apoptotic Jurkat cells were incubated with macrophages for 0 or 45 min at a 5:1 ratio. When pHrodo was phagocytized in the lysosome and pH decreased from neutral to acidic, its fluorescence intensity increased significantly. After incubation, the macrophages were washed twice and digested for flow cytometry or directly used for immunofluorescence imaging. In some groups, BMDMs were treated with MerTK CRISPR activation plasmid, ADAM17 siRNA as described before or 5 mM NAC.

For flow cytometry (FCM), macrophages cultured in 6-well dishes were digested with trypsin (Gibco, Franklin, Tennessee, USA) and washed. The cells were then suspended in FACS staining buffer (PBS containing 2% FBS and 1 mM EDTA) for analysis on a Beckman Coulter Cytoflex S flow cytometer. Efferocytosis was measured as the percentage of pHrodo-red and CMFDA-green double positive cells in all CMFDA-Green-positive cells.

For immunofluorescence photography (IF), macrophages in confocal dishes after co-culture with apoptotic cells were directly used for confocal laser scanning using a Zeiss LSM 900 laser scanning Confocal Microscopy. Apoptotic cells were phagocytosed and emitted strong fluorescence. Efferocytosis was measured as the percentage of pHrodo-red and CMFDA-green double positive cells in all CMFDA-green-positive cells.

### ROS detection

After efferocytosis, BMDMs in confocal dishes were washed three times to remove apoptotic cells. 5 µM Dihydroethidium (Yeason Biotechnology, Shanghai, China) was added to the confocal dishes and incubated for 60 min in incubators. The cells were then washed three times and scanned with a Zeiss LSM 900 Laser Scanning Confocal Microscope. The mean intensity value was obtained using the ZEN 3.0 application.

### Western blot

Tissues and cellular proteins were extracted using RIPA (Radio-Immunoprecipitation Assay) lysis buffer (Cell Signaling Technology, Danvers, Massachusetts, USA) supplemented with protease and phosphatase inhibitors (Cell Signaling Technology, Danvers, Massachusetts, USA). Proteins were subjected to sodium dodecyl sulfate-polyacrylamide gel electrophoresis (SDS-PAGE) and transferred onto a polyvinylidene 550 fluoride (PVDF) nitrocellulose membrane. Antibodies against MerTK, c-GAS, STING, phospho-STING (p-STING S365), TBK1, Phospho-TBK1 Ser172 (p-TBK1 S172), GAPDH (Cell Signaling Technology, Danvers, Massachusetts, USA), Cleaved caspase3 (Abcam, Branfold, Connecticut, USA), BCL-2, BAX (Proteintech, Rosemont, Illinois, USA), and ADAM17 (Novus Biologicals, Lehighton, Pennsylvania, USA) were used and incubated overnight at 4 °C. After 2 h of incubation with the appropriate horseradish peroxidase (HRP)-conjugated secondary antibody (Cell Signaling Technology, Danvers, Massachusetts, USA), bands were detected using Immobilon ECL Ultra Western HRP substrate (Vazyme Biotechnology, Nanjing, China), and images were acquired using a Vilber chemiluminescent imaging system.

### ELISA

Secretion of cytokines (IL-1β and IL-6) in serum was measured by ELISA, according to the manufacturer’s protocols (Thermo Fisher Scientific, Waltham, Massachusetts, USA).

### Data analysis

The results are presented as the mean ± SEM of three independent experiments. The Student’s *t* test and one-way ANOVA was used to analyze the differences among different groups. For all tests, a *P*-value < 0.05 (two-tailed) was considered significant.

## Data Availability

All data supporting the findings of this study are available within the manuscript and its supplementary information files.
